# SEMA6D Expression and Patient Survival in Breast Invasive Carcinoma

**DOI:** 10.1155/2015/539721

**Published:** 2015-04-20

**Authors:** Dongquan Chen, Yufeng Li, Lizhong Wang, Kai Jiao

**Affiliations:** ^1^Division of Preventive Medicine, Department of Medicine, University of Alabama at Birmingham, Birmingham, AL 35294, USA; ^2^Comprehensive Cancer Center, Department of Medicine, University of Alabama at Birmingham, Birmingham, AL 35294, USA; ^3^Research Division, Department of Genetics, University of Alabama at Birmingham, Birmingham, AL 35294, USA

## Abstract

Breast cancer (BC) is the second most common cancer diagnosed in American women and is also the second leading cause of cancer death in women. Research has focused heavily on BC metastasis. Multiple signaling pathways have been implicated in regulating BC metastasis. Our knowledge of regulation of BC metastasis is, however, far from complete. Identification of new factors during metastasis is an essential step towards future therapy. Our labs have focused on Semaphorin 6D (SEMA6D), which was implicated in immune responses, heart development, and neurogenesis. It will be interesting to know SEMA6D-related genomic expression profile and its implications in clinical outcome. In this study, we examined the public datasets of breast invasive carcinoma from The Cancer Genome Atlas (TCGA). We analyzed the expression of SEMA6D along with its related genes, their functions, pathways, and potential as copredictors for BC patients' survival. We found 6-gene expression profile that can be used as such predictors. Our study provides evidences for the first time that breast invasive carcinoma may contain a subtype based on SEMA6D expression. The expression of SEMA6D gene may play an important role in promoting patient survival, especially among triple negative breast cancer patients.

## 1. Introduction

Breast cancer (BC) is the second most common cancer diagnosed in American women and is also the second leading cause of cancer death in women [1, 2]. It is estimated that, in the developed world, one in eight women will develop breast cancer in her lifetime [3, 4]. BC lethality is mainly caused by metastasis, which accounts for approximately 90% of BC deaths [5–10]. Metastatic BC can be treated, sometimes for many years, but cannot be cured. BC primarily metastasizes to the bone, lungs, regional lymph nodes, liver, and brain, with the most common site being the bone. Research has focused heavily on BC metastasis for many years. Multiple signaling pathways, such as TGF*β*, Wnt, Notch, and EGF, have been implicated in regulating metastasis of BC cells [5–10]. However, our knowledge of regulation of BC metastasis is far from complete. Identification of new factors that play critical roles in driving/inhibiting metastatic progression is an essential step toward fully understanding BC metastasis and will also provide novel therapeutic targets/reagents against BC. Our labs have focused on Semaphorins (SEMAs), especially SEMA6D. SEMAs were implicated in immune responses, heart development, and neurogenesis [11–13] and recently in BC metastases [14–17].

Semaphorins were initially recognized as phylogenetically conserved neuronal guidance cues, and their critical regulatory roles in BC metastasis have rapidly emerged in recent years. Based on their sequence similarity, Semaphorins are classified into eight classes: classes 1-2 are found in invertebrates, classes 3–7 comprise the vertebrate Semaphorins, and class V is encoded by viruses. Class 2, 3, and V Semaphorins are secreted, while all other members are membrane tethered through a single transmembrane domain [17–25]. The signature structure of Semaphorins is the ~500 amino acid (aa) Sema domain, which is a variant of the *β*-propeller fold revealed from structural studies [22]. All Semaphorins, except class V, contain a PSI (Plexin-semaphorin-integrin) domain immediately to the C-terminal side of the Sema domain. Different classes of Semaphorins may also contain additional functional motifs.

Published studies regarding functions of Semaphorins in BC have mainly focused on class 3 secreted Semaphorins and SEMA4D [14–17]. Our recent bioinformatic analysis by using public datasets reveals for the first time the potential role of SEMA6D in regulating BC pathogenesis. The extracellular region of SEMA6D, which contains the Sema and PSI domains, can be released from the cell surface to act as a secreted cytokine with unknown molecular mechanisms [26, 27]. Thus, SEMA6D may act both locally through cell-cell contacts and more distantly through diffusion of its cleaved ectodomain. The primary receptors of SEMA6D are PLXNA1 and PLXNA4, which belong to the Plexin receptor family. The extracellular domains of Plexins interact with Semaphorins, while the C-terminal tails of Plexins mediate intracellular signal transduction. Unlike many signaling pathways (such as the TGF*β*/SMAD pathway), there is no “canonical” intracellular transduction cascade mediating activities of Semaphorin-Plexin signaling. Many intracellular signaling molecules, such as GTPase activating proteins, GTP/GDP exchange factors, and various tyrosine kinases, can be activated and/or inactivated by Semaphorin-Plexin signaling in a context-dependent manner [18–25].

In this study, we examine the public datasets from The Cancer Genome Atlas (TCGA), National Cancer Institute (NCI) for expression of SEMA6D along with genes that interact with SEMA6D. Other genes coregulated with SEMA6D were analyzed for their function, pathway, and potential as copredictors for BC patients' survival. We found 6-gene expression profile that can be used as such predictors. We also found that SEMA6D expression correlated with the cancer status of triple negative (TNBC) markers (ER, PR, and Her2 genes). The study shows the role of SEMA6D as potential survival predictor especially in TNBC patients.

## 2. Methods

### 2.1. Datasets

The Cancer Genome Atlas (TCGA) Data Portal was used to download breast invasive carcinoma (BRCA) samples (*n* = 1,100). The RNAseqV2 level 3 data, which includes fragments per kilobase of exon per million fragments mapped- (FPKM-) normalized gene level data, were used before statistics. In addition, idf and sdrf files were also downloaded for sample mapping and annotation. Clinical outcomes data were downloaded for correlation and survival analysis.

### 2.2. Gene Expression Data Analysis and Annotation

Gene-level normalized expression data were used in Partek Genomic Suite (PGS, St. Louis, MO) for additional normalization, statistics, and annotation. The analysis of variance (ANOVA) methods were used for group comparisons. False discovery rate (FDR) correction (Benjamini-Hochberg methods) was applied for multiple hypothesis testing purpose. Other statistical tools such as SAS (Cary, NC) and Ingenuity Pathway Analysis (IPA, Redwood City, CA) were used for pathway analysis and building gene-gene interaction network. Heatmap was generated by using hierarchical clustering methods after z-normalization.

### 2.3. Survival Analysis

A total of 140 patients with clinical outcomes data available (survival status, months of survival, demographics, and ER, PR, and HER2 status, etc.) were included in the analysis. Among significant genes after SEMA6D-high versus SEMA6D-low expression comparisons, we selected top 20 genes with the highest or lowest expression levels to correlate with clinical outcomes. Logarithm 2 based transformation of each gene was performed prior to any analysis. The correlation among these 20 genes was evaluated using Pearson correlation coefficient, and summary statistics were presented including mean with standard deviation, median, and range. Associations between level of genes and overall survival (OS) were assessed with Kaplan-Meier (K-M) curves and log-rank tests. Each gene was dichotomized as above or below median level of expression in the survival analysis. Significant association was determined at 5% type I error level. Multiple comparisons were not explicitly controlled for due to the small sample size and exploratory nature of the analysis.

## 3. Results and Discussion

Semaphorins, including members in subclass 3 and SEMA4D, have emerged as critical signaling molecules in regulating BC pathology [14–17]. The potential roles of other members of the Semaphorin family in BC have not been well addressed in the literature. Our ongoing studies suggest that SEMA6D plays a critical role in mediating Bone Morphogenetic Protein (BMP) signaling to regulate epithelial-mesenchymal-transition (EMT) by endocardial cells in developing hearts (Kai Jiao et al.'s manuscript in preparation). EMT is an essential step for initiating metastasis in BC and other cancers. We thus decided to apply a bioinformatic approach to examine the potential role of SEMA6D in BC tumorigenesis and progression using publically available datasets. After testing a few smaller datasets form Gene Ontology Omnibus (GEO), we chose to use a breast invasive carcinoma (BRCA) dataset from TCGA as it represents a major public data source with clinical outcomes information, such as overall survival after diagnosis. We divided all samples (*n* = 1,100) into three roughly equal sized groups based on SEMA6D expression (high, medium, and low). The comparison of SEMA6D-high versus SEMA6D-low expression group will reveal genes that are coregulated with SEMA6D.

**Figure 1:**
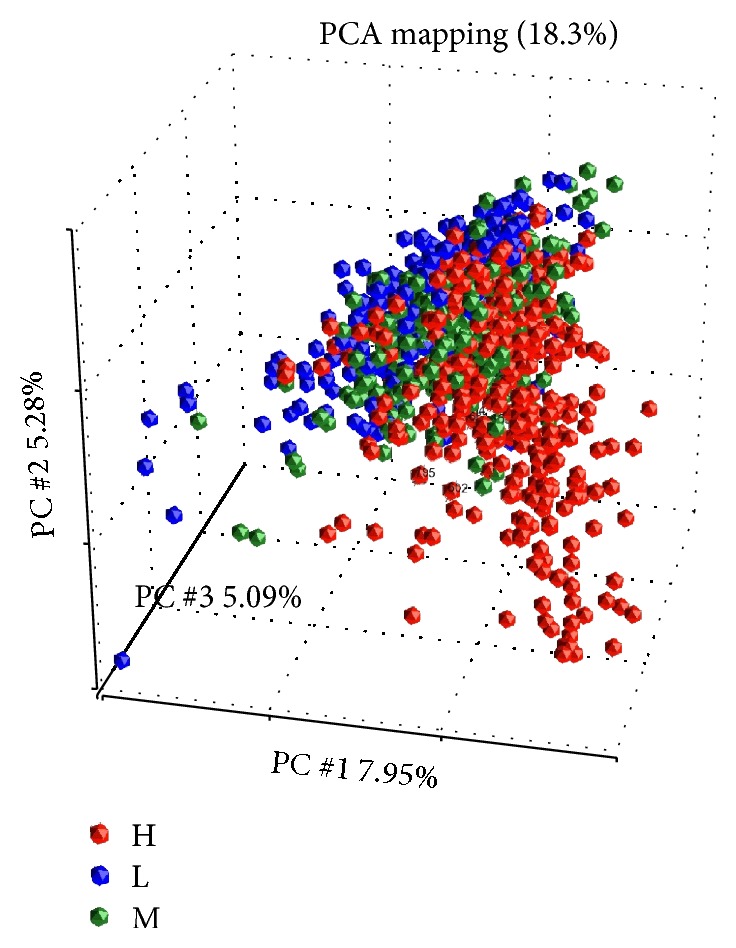
Principle component analysis (PCA) of all samples.

*Gene Expression Profile by Principle Components Analysis (PCA) and Hierarchical Clustering*. To examine overall gene expression profile and sample similarities, we perform the PCA analysis of all samples. As showed in Figure 1, the PCA showed a clear separation among SEMA6D-high (H), SEMA6D-medium (M), and SEMA6D-low (L) groups. This indicates different gene expression profiles among the three groups.

**Figure 2:**
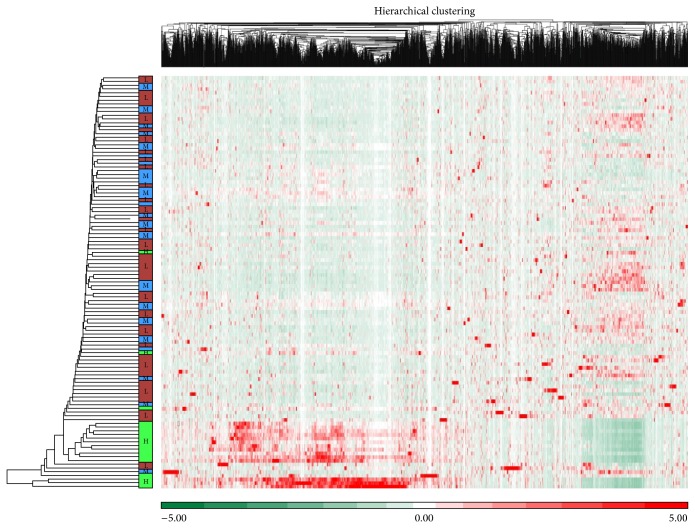
Hierarchical clustering of significant genes of SEMA6D-H versus -L expression. Genes (vertical: high expression in red and low expression in green) and samples (horizontal: SEMA6D-high in green, SEMA6D-medium in blue, and SEMA6D-low in brown) were clustered based on Euclidean dissimilarity matrix.

Based on the genes that are differentially expressed in SEMA6D-high versus SEMA6D-low expression groups, we then performed a hierarchical clustering (Figure 2) using log2-transformed normalized signal intensities. The observed separation of samples in each group (SEMA6D-high, SEMA6D-medium, and SEMA6D-low) indicates different expression profiles among these groups.

Consistent with the PCA analysis, the high SEME6D expression samples showed a congregation in the lower part of the figure, which indicates a clear separation of samples based on SEMA6D expression. In other words, BC samples may contain a subtype with high SEMA6D expression.

**Figure 3:**
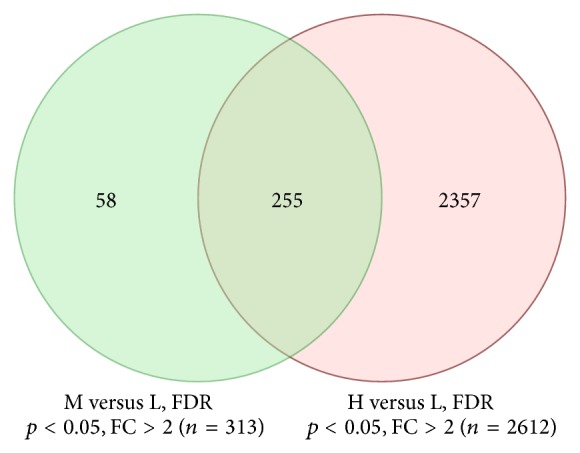
Number of significant genes between the two comparisons: H versus L and M versus L. FC: fold change.

We further examined SEMA6D levels by including SEMA6D-medium versus SEMA6D-low expression group comparison. As shown in the Venn diagram in Figure 3, 58 unique genes are significant between SEMA6D-medium and SEMA6D-low patients, while 2,357 genes are significant in SEMA6D-high versus SEMA6D-low expression comparison. This suggests that higher level of SEMA6D expression may lead to more significant changes, as evidenced by an increased number of genes with significant changes. This also suggests that the function of SEMA6D may be dependent on the expression level or that SEMA6D-induced functional effects are dose-dependent.

Among significant genes of SEMA6D-high versus SEMA6D-low comparison,* Gene Ontology (GO) analysis for biological processes* revealed that* multicellular organismal development and G-protein coupled receptor protein signaling pathway* are among the top changed GO biological processes (Table 1). It has been shown that SEMA6D may play a role not only during heart development [26, 27] but also during development of retina [28] and axon in zebra fish [29]. Our results support these findings and showed that SEMA6D played a major role in organismal development in addition to the G-protein coupled receptor signaling (Table 1).

The semaphorins and their receptors, the neuropilins and the Plexins, are constituents of a complex regulatory system that controls axonal guidance [30]. It was suggested that SEMA6D may bind to different receptor components and thus exert distinct functions during cardiac morphogenesis [26]. Our results suggested a broad function of SEMA6D to initiate signaling events that link to G-protein coupled receptor (GPCR) signaling (Table 1) and overall receptor activities (Table 2). GPCRs represent a super family of cell surface signaling proteins and play essential roles in cancer metastasis [2, 31, 32] and are one of the most promising targets of metastatic breast cancer therapy [33–37].

In cell adhesion, cell-cell interactions between cancer cells with endothelium determine the metastatic spread. There are two major cell adhesions, including selectin and integrin, and accumulating evidence confirms that tumor cell interactions through them actively contribute to the metastatic spread of tumor cells [38]. Our results suggest a pivotal role for SEMA6D in tumor metastasis especially receptor activities (Table 2) and GPCR signaling (Table 1).

**Figure 4:**
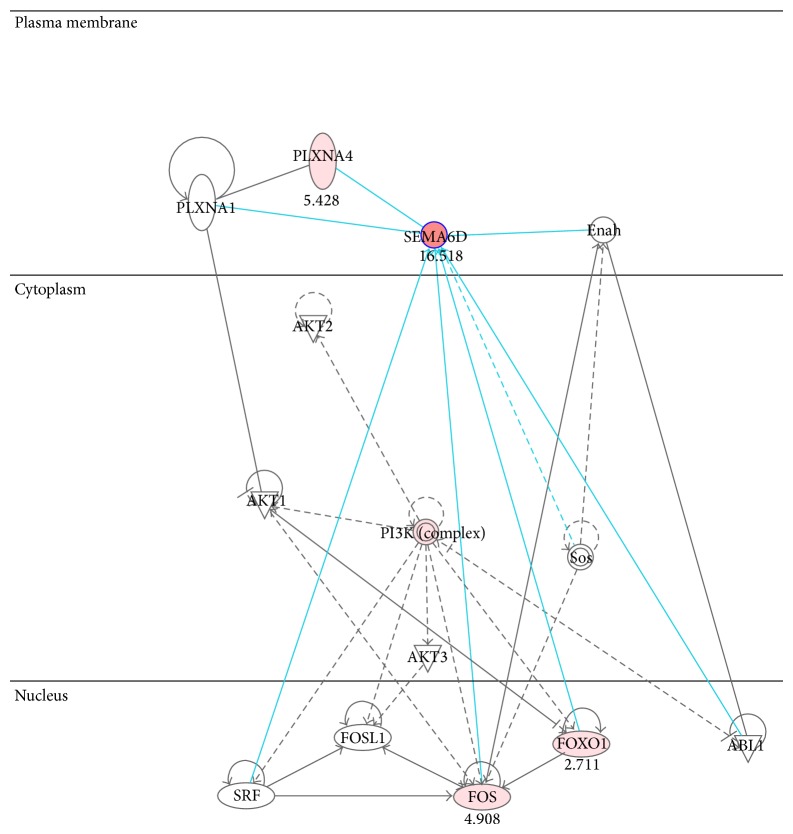
Activation of SEMA6D and transcription. The gene-gene interaction network was built based on direct interactions by using Ingenuity Pathway Analysis (IPA) suite. Color indicates increased (in red) expression when SEMA6D-high samples were compared with SEMA6D-low samples. The number indicated the fold changes of this comparison.

*The GO-molecular functions* also reveal that* receptor activity, sequence-specific DNA binding, and voltage-gated sodium channel activities* are among top affected molecular functions when SEMA6D level is high (Table 2). These results suggest that SEMA6D may initiate member receptors activation as a ligand. The gene-gene interactions among those genes that directly or indirectly interact with SEMA6D partially confirmed this hypothesis. As shown in Figure 4, elevated PLXNA4 may lead to an increase of SEMA6D expression and trigger transcriptions by the general transcription factors FOS and FOXO1. This may lead to a cascade of activations of membrane receptors including* G-protein coupled receptors* (Table 2).

As reported, Plexin-B1 is a receptor for the transmembrane semaphorin SEMA4D (CD100) [39], and PLXNA4 negatively regulates T lymphocyte responses [40]. It has been shown that SEMA6D induces NF-*κ*B transcriptional activity in nonmalignant mesothelial cells [30]. Two potential targets of SEMA6D, the general transcription factors FOS and FOXO1, were both increased in SEME6D-high patients. FOXO1 has been widely reported in tumor oncogenesis and metastasis [41–43]. This suggests an important role for SEMA6D in promoting general transcription through FOS coupled with FOXO1 as previously reported [44]. The balance of transcriptions of both tumor suppressors and oncogenes may be the key to understand the underlining mechanism.

*SEMA6D and Tumor Metastasis*. SEMA6D plays an important role in tissue development and differentiation, a process involving epithelial-mesenchymal-transition (EMT); it will be interesting to know if EMT-related genes are coregulated in SEMA6D-high patients. As shown in Table 3, major tumor metastatic promoter- (MMP-) 9 was dramatically reduced among SEMA6D-high samples, while several tumor metastatic promoters such as TGF-*β*-related factors, ZEB2, ZEB1, and GNG11, however, were elevated corresponding to a high level of SEMA6D. In addition, the expressions of all these genes (except for DSC2) are highly correlated with the expression of SEMA6D (Table 4). As SEMA6D was implicated in VEGF-dependent and anchorage-independent cell growth [30], we also included VEGF genes in the correlation analysis. High levels of correlations between SEMA6D level and VEGFs were found as well (Table 4) suggesting a role of VEGF family genes in mediating SEMA6D signaling.

MMP family proteins, especially MMP9, were suggested to be involved in the process of metastasis of breast cancer to the brain [45], CD147-mediated metastasis in MCF7 cells [19], TGF*β*-mediated signaling at the tumor-bone interface [46], and L2-mediated matrix remodeling in metastasis and mammary gland involution [47]. Decreased autocrine EGFR signaling in metastatic breast cancer cells inhibits tumor growth in bone and mammary fat pad through MMP9-dependent pathways [48]. By using an RNA interference approach, the reduced levels of MMP-9 mRNA and protein correlated with inhibited phenotype of tumor invasion and metastasis [14]. Our results are in line with these findings and suggest a tumor suppressor function for SEMA6D.

On the other hand, our results also showed an increased expression among SEMA6D-high samples of some important tumor promoters such as ZEB1/2, which had been reported to promote EMT by modulating Zeb1/2 and TGF*β* expression [49]. Our results thus strongly suggest that the balance between tumor suppressors and promoters is the key to understand the role of SEMA6D during EMT. Another explanation is that the increased expression of SEMA6D may be the results, not the cause ZEB1/2 changes and vice versa.

*SEMA6D Expression and Affected Signaling Pathways*. Although roles of SEMAs have been suggested in breast cancer [14–17], prostate cancer [50], and malignant mesothelioma [30], the underlying functional mechanisms including pathways are largely unknown. Nevertheless, SEMA6D has been reported to play a role in immune responses [12], NF-*κ*B signaling [30], and stromal expression of SEMA6D [50]. Our results implicated top canonical pathways (Table 5), which partially confirmed previous reports such as cAMP-mediated signaling in cervical cancer cell migration [51] and in lung cancer [52], G-protein coupled receptor signaling in breast cancer [34, 53], and adhesion and diapedesis in a breast cancer cell line [54]. Therefore SEMA6D may play multiple roles during these processes although additional studies may be needed to further delineate SEMA6D functions in these pathways.

**Figure 5:**
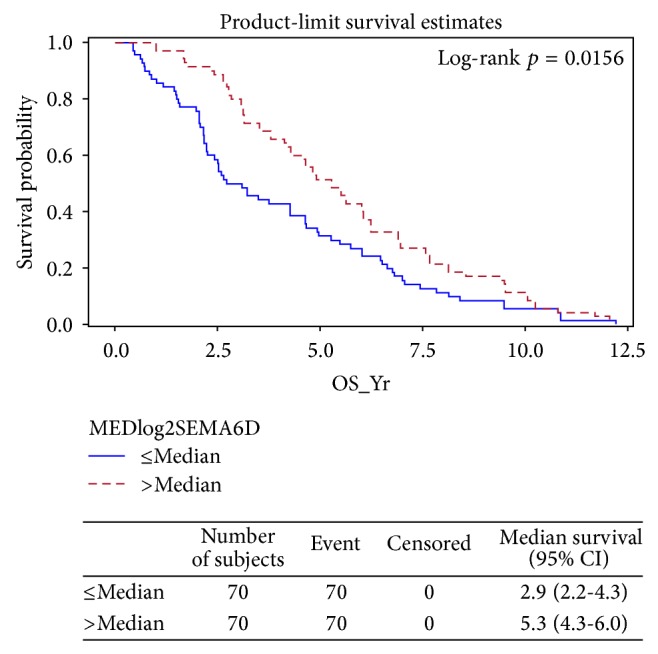
SEMA6D correlates with patient survival.

*SEMA6D Expression Correlates with Patients' Survival*. In order to determine if SEMA6D and SEMA6D-related genes are correlated with overall patient survival, we filtered the significant gene list after SEMA6D-high versus SEMA6D-low expression comparison by choosing the top 10 most upregulated genes and top 10 most downregulated genes and conducted a survival analysis by using K-M methods. We found that 6 candidate genes were significantly associated with overall survival. These genes are SEMA6D, CLEC9A, C10orf107, DONSON, CHAC1, and TUBA1C. Two more genes COL4A6 and CBX2 genes are in borderline to be significant. A summary of these 8 genes is listed in Table 6 and Figure 5.

In addition, increased expressions of SEMA6D, CLEC9A, COL4A6, and C10orf107 are associated with better survival while decreased expressions of DONSON, CHAC1, TUBA1C, and CBX2 also correlate to better survival. Figure 5 showed survival probability based on SEMA6D expression (≥median or ≤median expressions). Similar significant separation trends were also observed for both CLEC9A and C10orf107 as positive predictors and DONSON, CHAC1, and TUBA1C as negative predictors (data not shown).

**Figure 6:**
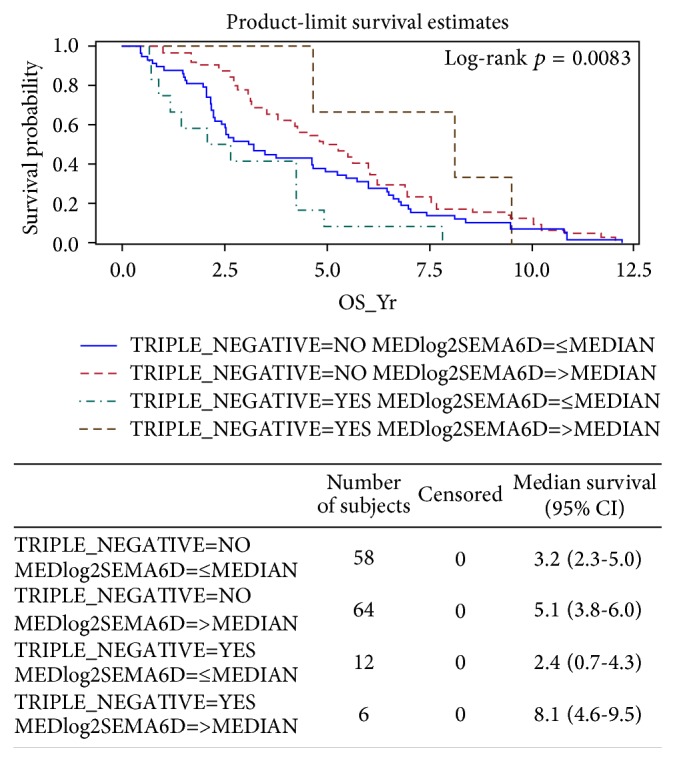
Interaction of SEMA6D with TNBC in patients' survival.

*Correlation of Expressions of SEMA6D and Other Genes with TNBC Status*. As only 30% of women with metastases survive five years and virtually all TNBC women will ultimately die of their disease despite systemic therapy [55], we further explore the role of SEMA6D in promoting survival in TNBC patients. We found not only that these genes associated with survival, but also that they are interacting with TNBC status (Yes or No) significantly for SEMA6D, for example, with a log-rank *p* = 0.0083, as shown in Figure 6. It is clearly shown that TNBC patients (Figure 6, SEMA6D-high in brown relative to SEMA6D-low in green) show larger survival differences as compared with non-TNBC patients (SEMA6D-high in red relative to SEMA6D-low in blue). Other genes such as CLEC9A (*p* = 0.0083) and C10orf107 (*p* = 0.0083) are similarly associated with TNBC status (data not shown).

These results strongly suggest that SEMA6D expression levels correlate with overall survival (Figure 5), especially in TNBC patients (Figure 6).

## 4. Conclusions

Our study provides evidences that breast invasive carcinoma (BRCA) may contain a subtype based on SEMA6D expression. The expression of SEMA6D gene may play an important role in promoting patient survival, especially among triple negative breast cancer (TNBC) patients.

## Figures and Tables

**Table 1:** Biological process: SEMA6D high versus low comparison.

Biological process	Enrichment score	Enrichment *p* value
Multicellular organismal development	34.34	1.22*E* − 15
G-protein coupled receptor protein signaling pathway	31.13	3.02*E* − 14
Cell adhesion	19.93	2.21*E* − 09
Nervous system development	19.92	2.23*E* − 09
Mitotic cell cycle	19.76	2.63*E* − 09
Cell division	18.62	8.22*E* − 09
Mitosis	18.54	8.90*E* − 09
M phase of mitotic cell cycle	18.29	1.14*E* − 08
Ion transport	17.24	3.27*E* − 08
Response to drug	16.08	1.04*E* − 07

**Table 2:** Molecular function: SEMA6D high versus low comparison.

Molecular function	Enrichment score	Enrichment *p* value
Receptor activity	22.90	1.14*E* − 10
Sequence-specific DNA binding	21.27	5.80*E* − 10
Voltage-gated sodium channel activity	20.10	1.87*E* − 09
Signal transducer activity	17.14	3.59*E* − 08
Calcium ion binding	16.56	6.43*E* − 08
Heparin binding	15.78	1.40*E* − 07
Voltage-gated ion channel activity	14.69	4.16*E* − 07
Receptor binding	14.43	5.43*E* − 07
G-protein coupled receptor activity	12.60	3.36*E* − 06
Sequence-specific DNA binding TF activity	11.60	9.14*E* − 06

**Table 3:** Expression of top EMT-related genes in SEMA6D-H versus L comparison.

Symbol	Description	*p* value (H versus L)	Fold change	Fold (description)
*MMP9 *	Matrix metallopeptidase 9 (gelatinase B, 92 kDa gelatinase, 92 kDa type IV collagenase)	0.163673	−3.61	H down versus L
*TMEM132A *	Transmembrane protein 132A	5.87*E* − 27	−2.21	H down versus L
*BMP7 *	Bone Morphogenetic Protein 7	2.85*E* − 05	−1.79	H down versus L
*DSC2 *	Desmocollin 2	8.55*E* − 07	−1.74	H down versus L
*HPRT1 *	Hypoxanthine phosphoribosyltransferase 1	7.82*E* − 46	−1.72	H down versus L
*KRT19 *	Keratin 19	1.44*E* − 15	−1.64	H down versus L
*SPP1 *	Secreted phosphoprotein 1	0.000141	−1.55	H down versus L
*PPPDE2 *	PPPDE peptidase domain containing 2	6.02*E* − 24	−1.48	H down versus L
*KRT7 *	Keratin 7	1.51*E* − 06	−1.48	H down versus L
*CDH1 *	Cadherin 1, type 1, E-cadherin (epithelial)	9.38*E* − 11	−1.46	H down versus L
*COL3A1 *	Collagen, type III, alpha 1	2.53*E* − 15	1.89	H up versus L
*MMP2 *	Matrix metallopeptidase 2 (gelatinase A, 72 kDa gelatinase, 72 kDa type IV collagenase)	3.74*E* − 25	1.97	H up versus L
*SNAI2 *	Snail homolog 2 (*Drosophila*)	8.28*E* − 24	2.08	H up versus L
*MITF *	Microphthalmia-associated transcription factor	1.33*E* − 42	2.08	H up versus L
*TCF4 *	Transcription factor 4	2.17*E* − 75	2.35	H up versus L
*AHNAK *	AHNAK nucleoprotein	5.98*E* − 57	2.37	H up versus L
*ZEB2 *	Zinc finger E-box binding homeobox 2	1.55*E* − 44	2.51	H up versus L
*ZEB1 *	Zinc finger E-box binding homeobox 1	2.14*E* − 73	2.67	H up versus L
*GNG11 *	Guanine nucleotide binding protein (G protein), gamma 11	1.31*E* − 54	3.30	H up versus L

**Table 4:** Correlation of SEMA6D with EMT gene expressions.

Symbol	*r*	*p* value
*GNG11 *	0.55	1.27*E* − 88
*ZEB1 *	0.55	1.38*E* − 87
*TCF4 *	0.53	5.32*E* − 82
*AHNAK *	0.52	7.08*E* − 79
*HPRT1 *	−0.49	1.12*E* − 68
*SNAI2 *	0.47	2.66*E* − 62
*ZEB2 *	0.46	1.53*E* − 59
*MITF *	0.45	5.23*E* − 55
*TMEM132A *	−0.43	1.05*E* − 49
*VEGFC *	0.38	9.27*E* − 39
*MMP2 *	0.33	5.47*E* − 30
*PPPDE2 *	−0.31	2.70*E* − 25
*MMP9 *	−0.27	7.18*E* − 20
*KRT19 *	−0.26	2.77*E* − 18
*SPP1 *	−0.22	8.70*E* − 14
*COL3A1 *	0.17	1.02*E* − 08
*VEGFA *	−0.15	2.76*E* − 07
*CDH1 *	−0.15	1.08*E* − 06
*VEGFB *	0.11	0.000235
*KRT7 *	−0.09	0.002199
*BMP7 *	0.07	0.015785
*DSC2 *	−0.04	0.188209

*r*: Spearman correlation coefficient, *n* = 1100.

**Table 5:** Canonical signaling pathway by SEME6D high expression.

Pathway name	*p* value	Ratio
cAMP-mediated signaling	2.27*E* − 09	53/222
G-Protein coupled receptor signaling	2.60*E* − 07	54/265
Granulocyte adhesion and diapedesis	2.73*E* − 07	41/176
Agranulocyte adhesion and diapedesis	1.52*E* − 06	41/187
Gas signaling	1.83*E* − 05	27/119

**Table 6:** Correlation of gene expression with patients' survival.

Variable	*N*	Mean	SD	Median	Min	Max	Log-rank *p*
*SEMA6D *	140	7.15	1.90	6.95	2.11	12.09	0.0156^*^
*CLEC9A *	127	2.12	1.93	2.36	−1.87	5.73	0.0308^*^
*COL4A6 *	139	5.65	2.67	5.79	0.18	10.77	0.0564
*C10orf107 *	134	2.50	2.03	2.67	−2.02	6.94	0.0019^*^
*DONSON *	140	7.99	0.91	7.87	6.36	10.46	0.0397^*^
*CHAC1 *	140	4.96	1.56	4.83	1.19	8.86	0.0003^*^
*TUBA1C *	140	11.61	0.99	11.74	9.39	14.51	0.0162^*^
*CBX2 *	140	7.71	1.93	7.36	2.81	11.60	0.0709

OS: overall survival, stratified by high (>medium) or low (<medium) expression ^*^ *p* < 0.05.

## Abbreviations

TNBC: Triple negative breast cancer
BRCA: Breast invasive carcinoma
BC: Breast cancer
SEMA6D: Semaphorin 6D
BMP: Bone Morphogenetic Protein
EMT: Epithelial-mesenchymal-transition
PCA: Principle component analysis
GO: Gene Ontology
TCGA: The Cancer Genome Atlas.
